# Comparative Study of Alterations in Tri-iodothyronine (T3) and Thyroxine (T4) Hormone Levels in Breast and Ovarian Cancer

**DOI:** 10.12669/pjms.306.5294

**Published:** 2014

**Authors:** Mahmood Rasool, Muhammad Imran Naseer, Kalsoom Zaigham, Arif Malik, Naila Riaz, Rabail Alam, Abdul Manan, Ishfaq Ahmed Sheikh, Muhammad Asif

**Affiliations:** 1 Mahmood Rasool, Center of Excellence in Genomic Medicine Research (CEGMR), King Abdulaziz University, Jeddah, Saudi Arabia.; 2 Muhammad Imran Naseer, Center of Excellence in Genomic Medicine Research (CEGMR), King Abdulaziz University, Jeddah, Saudi Arabia.; 3 Kalsoom Zaigham, Institute of Molecular Biology and Biotechnology (IMBB), The University of Lahore, Lahore, Pakistan.; 4 Arif Malik, Institute of Molecular Biology and Biotechnology (IMBB), The University of Lahore, Lahore, Pakistan.; 5 Naila Riaz, Institute of Molecular Biology and Biotechnology (IMBB), The University of Lahore, Lahore, Pakistan.; 6 Rabail Alam, Institute of Molecular Biology and Biotechnology (IMBB), The University of Lahore, Lahore, Pakistan.; 7 Abdul Manan, Institute of Molecular Biology and Biotechnology (IMBB), The University of Lahore, Lahore, Pakistan.; 8 Ishfaq Ahmed Sheikh, King Fahd Medical Research Center (KFMRC),; 9 Muhammad Asif, Department of Biotechnology and Informatics, (BUITEMS), Quetta, Pakistan

**Keywords:** Breast cancer, Ovarian cancer, Thyroid hormones, Tri-iodothyronine (T3), Thyroxine (T4)

## Abstract

***Objective*** ***:*** The present study was designed to investigate variations in the levels of thyroid hormones (T3, T4) in breast and ovarian cancers patients.

***Methods*** ***: ***A total 120 subjects were recruited (without thyroid history) divided into three groups; A, B and C. Group A as control with healthy individuals. While group B and group C were consisting of breast cancer and ovarian cancer patient respectively. Blood samples (5 ml) were taken and analyzed to estimate the levels of serum T3 (tri-iodothyronine) and T4 (thyroxin) hormones.

***R*** ***esults*** ***:*** Statistically significant difference (P=0.000* and P=0.017*) was obtained among all groups. A significant increase in T3 (P=0.000*) and T4 (0.005*) levels was observed among breast cancer patients as compared to healthy controls. While for ovarian cancer patients conflicting results were found for T3 and T4 levels in the serum i.e. insignificant difference was found in T3 (P=0.209) and T4 (P=0.050) as compared to control. Our results showed that in the breast cancer and ovarian cancer patients the thyroid hormone (T3 and T4) level has been altered from the normal ranges as compared to the normal healthy individuals.

***Conclusion*** ***:*** We conclude that hyperthyroidism has profound effects on breast cancer and ovarian cancer cells proliferation.

## INTRODUCTION

Thyroid hormone plays an imperative role in the regulation of cellular metabolism, proliferation and differentiation.^1^ There are two basic forms of thyroid hormone: T4 (3,5,3',5'-tetraiodothyronine) and T3 (3, 3',5-triiodothyronine). The two forms of thyroid hormone (T3, T4) are produced and secreted by the follicular cells of the thyroid gland.^2^

 Thyroid hormones mediate their action on different types of cells (plasma membrane, nucleus, cytoplasm and in the mitochondria) in different ways. For example, the biological activities of T3 are regulated by thyroid hormone nuclear receptors (TRs) via transcriptional regulation. There are two different TR genes in humans; α and β which encode for different T3-binding receptor isoforms (α1, β1, β2 and β3). Any mutations in the *TRβ* gene decrease the sensitivity of the target tissue of the thyroid hormone.^3^ However, the transcriptional activity of T3 can be upregulated by many factors which include host of nuclear co-regulatory proteins, the type of thyroid hormone response elements situated on the promoter sites of the T3 target genes as well as the developmental- and tissue dependent expressions of TR isoforms. These co-regulatory proteins consist of corepressors and coactivators, which either repress or activate the transcription, respectively.^3^

 Autoimmune thyroid diseases affect the thyroid hormone production that results in either hypothyroidism or hyperthyroidism. They cause two opposing clinical syndromes, Hashimoto’s thyroiditis (HT) and Graves’ disease (GD).^4^^,^^5^

 Thyroid disease has been associated with many diseases including breast cancer.^3^^,^^6^ It is a well-known fact that breast cancer is a hormone-dependent neoplasm. There have been reports on the clinical association between breast cancer and thyroid diseases but at the same time there are many contradictory results reported in literature which do not verify the clinical association between these two diseases. Geographical distinction in the incidence of breast cancer has been ascribed due to differences in intake of iodine in diet, and an effect of iodine on breast was also suggested. Our results signify an increased occurrence of thyroid diseases (both autoimmune and nonautoimmune) in breast cancer patients.^6^

 The concept that thyroid hormone may act as a growth factor for the breast cancer has been reported by various scientists.^7^ The thyroid hormone receptors and steroids belong to the same family with similar molecular structure, but having different transcriptional functions which define their genomic actions.^1^

Thyroid hormones, T3 and T4 play a role in tumor cell proliferation and angiogenesis via binding to αVβ3 receptor at plasma membrane. Tetraiodothyroacetic acid (tetrac), displays an anti-proliferative activity by blocking the proangiogenic and proliferative actions of T3 and T4. In nucleus many transactivator proteins like estrogen receptor-α (ERα), thyroid hormone receptor-β1 (TRβ1) and signal transducer and activator of transcription-1α (STAT1α) are phosphorylated by translocated pMAPK. These downstream phosphorylated transactivator proteins start gene transcription of fibroblast growth factor factor (bFGF), (that induces thyroid hormone-induced angiogenesis). These also start transcription of other proliferation factors which are important for cell division of tumor cells.^8^

 On the other hand ovarian cancer is also a hormone-dependent neoplasm like breast cancer. Ovarian cancer develops when a mutation or genetic change occurs in the cells on the surface the ovaries or in the fallopian tubes that leads to uncontrolled cell growth which may often metastasize.^9^ In hyperthyroidism where there is a higher concentration of thyroid hormones (T3 and T4), circulating in the blood and reaches to the ovarian tissue, causes its inflammation. The ovarian surface epithelial (OSE) cells exhibit the receptor for thyroid hormone and estrogen hormone. T3 exerts direct inflammatory effects on the ovarian epithelial cells. When T3 binds to its receptor at OSE, it increases the expression of ERα and mRNA which mimic the action of estrogen receptor and encodes the ER isoforms. These isoforms are strongly associated with ovarian cancer. It suggests a possible link between hyperthyroidism and ovarian cancer.^10^

 The role of thyroid hormones in the development and differentiation of normal breast tissue is a well known fact. However, the clinical association between the breast cancer and thyroid diseases is controversial. Although some of the studies do not verify this association but there are significant number of studies which established the clinical association. In addition to this, there are some studies which suggested a possible link between ovarian cancer and thyroid diseases.^6^^,^^7^^,^^10^^-^^12^ This study is perhaps the first from Lahore, Pakistan to elucidate the role of thyroid hormone abnormalities (autoimmune or non-autoimmune disorders) and to correlate them with breast and ovarian cancers with respect to T3 and T4 (having genetic and/or environmental influences). Moreover, if it is established as expected, then the effect of interferon received by hepatitis C patients,^13^ particularly in females who suffer from thyroid disease (as side effect of interferon) may be linked to breast and ovarian tumor/cancer.

## METHODS

The blood (5 ml) from breast and ovarian cancer patients were collected from pathology Laboratory at INMOL (Institute of Nuclear Medicine and Oncology) hospital Lahore. The samples of healthy individuals (control) were collected from different cities of the Punjab i.e. Lahore, Gujranwala and Faisalabad etc with age limits of 18 to 70 years old.

***Experimental design*** *: *A total 120 subjects were selected for the present study. They were divided into three groups as A, B and C (Table-I). Group A consisted of normal healthy individuals (control). Groups B and C consisted of histopathologically confirmed breast cancer and ovarian cancer patients respectively without their thyroid history (i.e. patients having symptomatic for thyroid disorder).

***Sample collection and processing*** *: *Blood samples (5 ml) from female subjects were collected in a clot activator vacutainer blood collection tube (BD^®^). After clot formation in the blood tubes the serum was separated out by centrifugation at 4000 rpmfor 10 min.

***Sample storage: ***The serum was then separated from the clotted blood and transferred into a sterile glass tubes/eppendorf tubes. It was properly labeled with patient’s identification number and was stored at 4°C.

***Estimation of serum thyroid hormone levels tri-iodothyroxine (T3) and thyroxine (T4)*** ***:*** Serum thyroid hormones (T3, T4) level was estimated by competitive radioimmunoassay.^14^^,^^15^

***Statistical Analysis: ***The data thus obtained was subjected to statistical analysis for the determination of significance by using ANOVA (Analysis of Variance).

## RESULTS

Overall statistically significant difference (P<0.05) was observed in mean values of serum thyroid hormones (T3, T4) level in all groups, A (control), B (Breast cancer) and C (Ovarian cancer) as compared to control group.

 Multiple comparisons (Table-II) indicated that there was a significant difference (P<0.05) in serum T3 levels in CA breast cancer patients when compared to control and CA ovarian cancer patients while an insignificant difference (P>0.05) was observed in ovarian cancer patients as compared to control group. Significant difference (P<0.05) was observed in serum T4 levels in CA breast and CA ovarian patients when compared to control.

 A positive significant (P<0.05) correlation was observed between T3 and T4 levels among breast and ovarian cancer patients (Table-III), but there was no significant correlation for the levels among the control group.

## DISCUSSION

As mentioned in introduction, this study is the stepping stone to elucidate the role of thyroid hormone abnormalities and to correlate them with breast and ovarian cancers with respect to T3 and T4. Significant increase in T3 and T4 levels were seen among breast cancer patients as compared to control (P<0.05) which was in agreement with work of^11^^,^^16^ where it has been observed that hyperthyroidism is associated with breast cancer patients with significantly higher T3 and T4 values and lower TSH levels which is suggesting a possible tumor growth promoting effect caused by this imbalance. They evaluated that in iodine deficient women the administration of thyroid hormone seems to elevate the chance of developing breast cancer. The occurrence of breast cancer was twice higher among hypothyroid women who were on supplemental thyroid hormone when compared to women not on thyroid hormone supplements. It has been reported that the incidence of breast cancer was almost half in women taking thyroid hormone for 5 years (10%) in comparison to women who have been taking thyroid hormone more than 15 years (19.5%).^17^

Significant increase (P<0.05) in T4 level was observed in ovarian cancer patients as compared to control,^10^^,^^12^ where associations between hyperthyroidism and ovarian cancer has been observed which was a novel observation. Ovarian cancer is hormone dependent inflammation and the most frequent reason of hyperthyroidism is autoimmune inflammation of the thyroid gland.

However, no significant difference was observed in T3 level in ovarian cancer patients when compared to controls which may be attributed to its lower concentrations in the circulation as well as due to its very short half life (5.3 days) as compared to T4 (5.7 days).^18^ It was inferred from the highly positive correlation between T4 and T3 levels in ovarian cancer as well as among breast cancer patients. This showed a definite relationship between these two hormones (T3 and T4) as T4 is transformed to T3 within the target cells with the help of deiodinases (types of deiodinases, D1, D2, D3).^19^

**Table-I:** Estimation of serum T3 and T4 levels

	**Group A**	**Group B**	**Group C**	**P value**
**T3 **(nm/L)	2.01 ± 0.39	2.73 ± 0.82	2.25 ± 0.71	0.000*
**T4 **(nm/L)	117.65 ± 25.35	146.91 ± 42.92	137.68 ± 37.41	0.017*

**Table-II:** Multiple comparisons of T3 and T4 levels among the groups

	**Control Group**	**Cancer Group**	**P value**
T3	Control	CA Breast	0.000
		CA Ovary	0.209
T4	Control	CA Breast	0.005
		CA Ovary	0.050

**Table-III:** Pearson correlation (two-tailed) between t3 and t4 levels among breast and ovarian cancer patients

	**T3 Control**	**T3 Ovarian**	**T3 Breast**	
**T4 Control**	0.373	0.008	0.138	**r-value**
	0.105	0.974	0.563	***p*** **-value**
**T4 Ovarian**	-0.520	0.502	0.206	**r-value**
	0.019*	0.000**	0.151	***p*** **-value**
**T4 Breast**	0.596	0.047	0.533	**r-value**
	0.006**	0.746	0.000**	***p*** **-value**

**r-value**: correlation coefficient.

*, **: Significant at 0.05 and 0.01 respectively

Geographical variations in the rates of endometrial, breast, and ovarian cancers are inversely correlated with iodine intake in the diet. Endocrinological studies^20^^-^^22^ have indicated that a low dietary iodine intake may result in a state of increased gonadotrophin stimulation which in turn leads to a hyperestrogenic state that gives rise to increased production of estradiol and estrone as well as a relatively low estriol to estrone plus estradiol ratio. The difference in the endocrine state may increase the chance of endometrial, breast and ovarian cancers while the increase in dietary intake of iodine may decrease the risk of the above mentioned cancers.^23^

Since significantly elevated levels of T3 and T4 were observed in breast cancer patient, it can be concluded that manifestation of breast cancer is associated with serum thyroid hormones (T3, T4) levels. However, for ovarian cancer, there was no significant difference in the level of T3 but not T4 levels when compared to control. A hyperthyroidism has profound effects on breast cancer and ovarian cancer cells proliferation since significant alterations in amount of thyroid hormone (both T3 and T4) in breast cancer and T4 in ovarian cancer patients has been reported. This study would be of significant importance in screening of these patients related to the risk of these cancers (breast and ovarian) with other molecular biomarkers of tumor growth.

## CONCLUSION

This study has significant impact on screening of female patients for breast and ovarian cancers. Estimation of T3 and T4 levels along with the panel of other biomarkers may have profound effect in screening of these patients. Since there have been contradictory reports regarding correlation of hyperthyroidism and breast cancer, this study is an addition in this regard and suggests the correlation between hyperthyroidism (higher T3 and T4 levels) and breast cancer. In addition this study also provides basis and opens new dimensions in screening of ovarian cancer patients using T4 level estimation along with other biomarkers. Since interferon therapy may lead to thyroid dysfunction, this study also suggests possibility of progression of breast and ovarian cancer in patients receiving interferon therapy. Hence patients receiving interferon therapy shall also be screened for these cancers. Over all this study has impeccable impact on screening of breast and ovarian cancer patients having thyroid gland dysfunction.
